# Temporal Trends in X-Ray Exposure during Coronary Angiography and Percutaneous Coronary Intervention

**DOI:** 10.1155/2020/9602942

**Published:** 2020-08-31

**Authors:** Cedric Davidsen, Kirsten Bolstad, Ellisif Nygaard, Kjell Vikenes, Svein Rotevatn, Vegard Tuseth

**Affiliations:** ^1^Department of Heart Disease, Haukeland University Hospital, Bergen, Norway; ^2^Department of Oncology and Medical Physics, Haukeland University Hospital, Bergen, Norway; ^3^Department of Clinical Medicine, University of Bergen, Bergen, Norway

## Abstract

**Background:**

Percutaneous coronary intervention exposes patient and staff to ionizing radiation. Although staff only receive a small fraction of patient dose through scatter radiation, there are concerns about the potential health effects of repeated exposure. Minimizing both patient and occupational exposure is needed.

**Objective:**

This article investigates patient and operator X-ray exposure over time in coronary intervention in relation to upgraded X-ray equipment, improved shielding, and enhanced operator awareness.

**Materials and Methods:**

Data regarding irradiation time, patient dose, and patient characteristics were extracted from the Norwegian Registry for Invasive Cardiology (NORIC) for procedures performed from 2013 to mid-2019. Personal operator dosimetry records were provided by the Norwegian Radiation and Nuclear Safety Authority. Improved operator shielding and awareness measures were introduced in 2018.

**Results:**

In the period 2013 through June 2019, 21499 procedures were recorded in our institution. Mean dose area product (DAP) for coronary angiography decreased 37% from 2981 *μ*Gy·m^2^ in 2013 to 1891 *μ*Gy·m^2^ in 2019 (*p* < 0.001). For coronary intervention, DAP decreased 39% from 8358 *μ*Gy·m^2^ to 5055 *μ*Gy·m^2^. Personal dosimetry data indicate a 70% reduction in operator dose per procedure in 2019 compared to 2013. The most pronounced reduction occurred after improved radiation protection measures were implemented in 2018 (−48%).

**Conclusions:**

This study shows a temporal trend towards considerable reduction in X-ray doses received by the patient and operator during cardiac catheterization. Upgraded X-ray equipment, improved shielding, and enhanced operator awareness are likely contributors to this development.

## 1. Introduction

Each year, approximately 450,000 percutaneous coronary intervention procedures are performed in the United States [1]. During these procedures, the acquisition of X-ray images exposes patient and staff to ionizing radiation. The potential harmful effects can be divided in two categories. Deterministic effects occur at a certain threshold of absorbed dose such as skin erythema, cataract, or epilation. Stochastic effects are random effects due to radiation-induced DNA damage that may increase the lifetime risk of developing malignancies. Most data on stochastic effects are derived from survivors from the Hiroshima bomb where high exposures lead to increased risk of cancer [2]. Although the effect of low-dose radiation is still debated [3, 4], there are growing concerns among interventional cardiologists about the potential harmful health impact of long-term exposure to scatter radiation [5–9]. The International Commission on Radiation Protection recommends that X-ray exposure should be kept as low as reasonably achievable with recommended dose limits of <20 millisievert (mSv) effective dose per year for staff working in the cardiac catheterization lab (cath lab) [10]. For comparison, the global average natural background radiation exposure is 2.4 mSv/year [11], and a CT-scan typically exposes a patient to effective doses in the magnitude of 1–10 mSv [12].

During coronary angiography and percutaneous coronary intervention (PCI), staff wear a personal dosimetry badge, which measures exposure to scatter radiation. Operator dose is proportional to patient dose, which decreases with the square of the distance from the radiation source and can be further effectively reduced by shielding. Shielding equivalent to 0.5 mm lead (Pb) reduces the transmitted scatter radiation by >90% [13]. A combination of table- and ceiling-mounted shields are used to reduce staff exposure. They are, however, bulky and cumbersome, and gaps between the different shielding components tend to appear during the procedure when the operator is shifting table position, height, and angle of C-arm. Thus, operator awareness is crucial for optimal use. In addition, staff exposed to >1 mSv year are required to wear lead aprons. These, however, do not protect the extremities and are heavy, uncomfortable, and can lead to orthopedic problems [5].

Patient effective dose is determined by several factors, and hence more complex to quantify. Some factors are easily quantifiable, such as patient weight and exposure time. Others vary during the procedure—irradiation field size, pulse rate, collimation, and angle of the X-ray tube. As effective dose is not directly available with existing equipment, the dose area product (DAP) is most frequently used to document patient exposure. DAP is the product of dose expressed in gray multiplied by the area irradiated. Common units are microgray meters squared (*μ*Gy·m^2^) and gray centimeters squared (Gy·cm^2^). An estimate of patient effective dose in mSv can also be calculated by multiplying DAP with a conversion factor [14].

In this study, we aimed to investigate patient and operator X-ray exposure over time in coronary intervention in relation to upgraded X-ray equipment, improved shielding, and enhanced operator awareness.

## 2. Materials and Methods

The study uses registry data in retrospective exploratory analyses of patient and operator radiation exposure during coronary angiography and PCI. Our data are limited to procedures performed in three full-time cardiac cath labs at Haukeland University Hospital, Norway, from January 2013 to June 2019. The Norwegian Registry for Invasive Cardiology (NORIC), which records nearly every coronary procedure performed in Norway, provided data on patient characteristics and procedural details such as DAP, irradiation time, and operator. The study was approved by the local ethics committee prior to data extraction and analysis.

The Norwegian Radiation and Nuclear Safety Authority provides a personal dosimetry subscription service including personal thermoluminescent dosimeter badges, which record both H10 and H0.07 (dose equivalent to the soft tissue at depths of 10 mm and 0.07 mm, respectively). The badges are worn by all cath lab staff and returned for dosimetry readings every two months. These readings were used to assess occupational exposure. Operator dose in this article refers to H10 measurements of the operators. Dosimetry data from nurses working in the cath labs were not included in the analysis since NORIC does not document which nurses are present during the procedures.

Between 2013 and 2019, two of the three cath labs were upgraded with new C-arms, and improved shielding measures were introduced. In January 2016, a Siemens Axiom Artis dFC from 2005 was replaced with a new Siemens Artis Q. In September 2018, a biplane Siemens Axiom Artis dBC from 2006 was upgraded to a Philips Azurion7 B12/12 biplane. The third cath lab, a Philips Allura Xper FD10 C, installed in October 2009, did not undergo any upgrades. Additionally, the transparent ceiling-mounted protective shields in all three cath labs were replaced with larger panels with lead curtains on the lower side in 2016. The same year, real-time dosimetry (Philips DoseAware®) was installed, providing instant feedback on radiation exposure during the procedures. In 2018, a program to increase awareness on radiation protection with focus on the importance of operator shielding [15, 16] was initiated. This led to the introduction of routine use of a 40 × 75 cm pelvic lead shield being placed directly adjacent to the ceiling arm mounted transparent shield. In large patients or complex procedures, an additional wheel-mounted side screen was used at operator's discretion. Real-time dosimetry measurements performed during clinical cases to validate the approach-suggested substantial benefit, and improved shielding was generally implemented by staff.

Statistical analyses were performed in RStudio: integrated development for *R* version 1.1.456 (RStudio, Inc., Boston, MA). Graphics were produced with the ggplot2 package 2.2.1. Between-group differences were evaluated using Student's *t*-test for continuous variables and the chi square test/Fisher's exact test for categorical variables. Temporal trends were evaluated with linear regression for continuous variables and logistic regression for categorical variables. Impact of C-arm upgrade was analyzed with the Kruskal–Wallis test by ranks (one-way ANOVA on ranks), followed by the Wilcoxon–Mann–Whitney test for pairwise comparison for each lab before and after upgrade. The associations between observed patient DAP and irradiation time, patient weight, time elapsed from start of study, upgrades of the X-ray equipment, and whether PCI or angiography was performed were evaluated using multiple linear regression.

### 2.1. Missing Data and Data Cleaning

Data on patient sex and age are automatically derived and calculated in NORIC based on information in the national identity number and the date of procedure. Height, weight, irradiation time, and DAP are manually entered in the registry. 1.3% of patient weight and 2.3% of patient height values were missing. Extreme values (height <140 and >200 cm and weight <40 and >150 kg) were manually checked and corrected in case of apparent typing error. For the analysis of irradiation time and DAP, only complete cases that included both variables were included. Extreme values (DAP < 200 or >80.000 *μ*Gy·m^2^ and irradiation time <30 or >10800 s) were excluded as they most probably represent input error or extreme procedures that do not represent the general trend. An additional filter on dose per second (<0.7 or >50 *μ*Gy·m^2^/s) was added to exclude observations with mismatch between dose and irradiation time as these most probably represent input error. A total of 3.7% of irradiation time and DAP values were excluded from primary analysis. For the analysis of the ratio between yearly operator dose in mSv and patient DAP, a complete dataset was required, and missing DAP values were imputed using the MICE package version 3.7.0 (Multivariate Imputation by Chained Equations in R). Five imputed datasets were created using predictive mean matching. Mean DAP per procedure was estimated in each imputed dataset separately and then combined using Rubin's rules.

## 3. Results

### 3.1. Patient Characteristics

In the total material, 70.5% of the patients were male. The proportion did not change significantly from 2013 to 2019. Mean patient age was 66.8 years, and females were on average 4.3 years older than males (65.6 vs 69.9 years, *p* < 0.001). From 2013 to 2019, mean age increased with 1.2 years (*p* < 0.001). Age increase was slightly larger for men (1.5 years) than women (0.7 years). Mean (median) body mass index (BMI) for all patients was 27.2 (26.7) and higher in men (27.5) than in women (26.6, *p* < 0.001). From 2013 to 2019, mean BMI increased from 27.0 to 27.4 (*p* < 0.001), mostly driven by a BMI increase in men (+0.6, *p* < 0.001), whereas there was no significant change in female BMI. A complete list of patient characteristics is available in Table 1.

### 3.2. Procedural Characteristics

**Figure 1:**
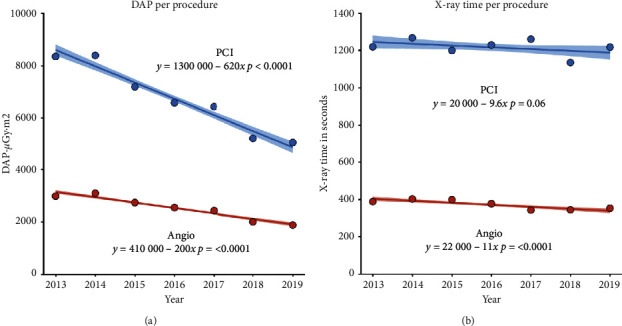
Trends in dose area product (DAP) and irradiation time per procedure. Between 2013 and 2019, there was a trend towards reduced DAP per procedure (a). On average, yearly reduction in DAP per procedure was 620 *μ*Gy·m^2^ in PCI (*p* < 0.001) and 200 *μ*Gy·m^2^ in coronary angiography (*p* < 0.001). Dots represent mean DAP per procedure. The linear regression line and standard error were calculated on the entire dataset (*n* = 20709). For irradiation time (b), there was a small but significant trend towards a reduction in irradiation time of 11 seconds per procedure per year in angiography (*p* < 0.001).

A total of 21499 procedures were recorded in NORIC from 2013 to June 2019. 54% of procedures were diagnostic coronary angiography and 46% PCI. Between 2013 and 2019, the proportion of PCI increased from 41.7% to 47.9% (*p* < 0.001). Mean DAP was higher in PCI than in coronary angiography (6793 vs 2574 *μ*Gy·m^2^, *p* < 0.001) and decreased in both groups (−39% and −37%, respectively, Table 2, Figure 1(a)). Mean irradiation time was longer in PCI (1217 vs 373 seconds, *p* < 0.001) and decreased for coronary angiography (−9%) but not for PCI (Table 2, Figure 1(b)). The ratio of DAP divided by irradiation time was calculated to evaluate trends in patient exposure corrected for changes in irradiation time per procedure and decreased both for coronary angiography (−30%) and PCI (−39%)

### 3.3. Influence of Weight and Age on Irradiation Time and Patient Dose (DAP)

**Figure 2:**
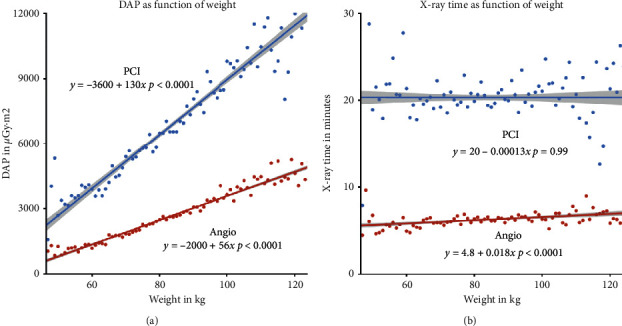
Influence of patient weight on dose area product (DAP) and irradiation time. Patient weight had a strong correlation to DAP (a). The dots represent the mean DAP for all patients with a specific weight. The linear regression line and standard error were calculated on the entire dataset (*n* = 20 709). Each additional kilogram patient weight leads to an increase in 130 *μ*Gy·m^2^ in PCI and 56 *μ*Gy·m^2^ in coronary angiography (*p* < 0.001).Patient weight had only a minor influence on irradiation time (b).

Increased patient weight was correlated to higher DAP per procedure (Figure 2(a)). In patients weighing 50–60 kg, mean DAP was 1189 *μ*Gy·m^2^ in angiography and 3722 *μ*Gy·m^2^ in PCI. In patients 100–110 kg, mean DAP was to 4061 *μ*Gy·m^2^ in angiography and 9915 *μ*Gy·m^2^ in PCI. Patient weight had only a minor effect on irradiation time (Figure 2(b)). There was a small trend towards increased irradiation time with increasing patient weight in coronary angiography, but no such trend was present in PCI.

Patient age impacted irradiation time, and older patients had a trend towards longer procedures (supplementary Figure 1(a)). In patients aged 50–55 years, mean irradiation time was 5 minutes (*m*) 24 seconds (*s*) in angiography and 17 m 36 s in PCI. In patients aged 80–85 years, mean irradiation time increased to 7 m 18 s in angiography (+35%) and 22 m13 s (+26%) in PCI. Despite increasing irradiation time with increasing patient age, there was no trend towards higher DAP per procedure in older patients (supplementary Figure 1(b)). This may be explained by lower patient weight (supplementary Figure 1(c)) in both older males and females, as well as a larger proportion of female patients as patient age increases (supplementary Figure 1(d)).

### 3.4. Impact of the C-Arm Model on Patient Dose (DAP)

**Figure 3:**
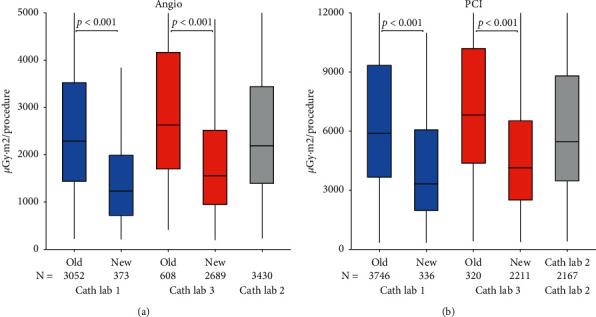
Influence of C-arm upgrade on dose area product (DAP). Boxplot representing DAP per procedure in the three cath labs at our institution before and after C-arm upgrade. Newer C-arms were associated with lower DAP per procedure both in isolated coronary angiography (a) and in PCI (b). Cath Lab 1, Siemens Axiom Artis dBC installed in 2006 was replaced with a Philips Azurion 7 B12/12 in 2018, which led to a 50% reduction of mean DAP in angiography and 41% in PCI (*p* < 0.001). Cath Lab 3, Siemens Artis Axiom dFC installed in 2005 was replaced in 2016 with a Siemens Artis Q. Mean doses on the new lab was 28% lower in angio and 32% lower in PCI (*p* < 0.001). Cath Lab 2, Philips Allura Xper FD10C installed in 2009 did not undergo an upgrade.

Between 2013 and 2019, two out of three cath labs were upgraded. There was significant variation in DAP per procedure between cath labs. Newer labs had on average lower doses both for angiography and PCI (Figure 3, supplementary Table 1). In January 2016, a Siemens Axiom Artis dFC monoplane from 2005 was replaced with a Siemens Artis *Q*, which leads to a decrease in mean DAP of 40% for angiography from 3333 (median 2630) *μ*Gy·m^2^ to 1978 (median 1553) *μ*Gy·m^2^ (*p* < 0.001). In September 2018, a Siemens Axiom Artis dBC biplane from 2006 was upgraded to a Philips Azurion 7 Biplane, and the mean DAP for angiography was reduced with 50% from 3303 *μ*Gy·m^2^ (median 2294) to 1650 *μ*Gy·m^2^ (median 1230, *p* < 0.001). Similar decreases were observed for PCI.

### 3.5. Multivariable Analysis of Factors Influencing Patient Dose (DAP)

A multivariable linear regression model was created evaluating patient DAP as function of days elapsed since 1^st^ January 2013, procedure type (angiography or PCI), patient weight, irradiation time, and upgrade of two of the cath labs (categorical variable as before/after upgrade of the cath labs). The linear regression equation retained significance for all tested variables with adjusted R-squared 0.6239, and *p* value for the model <0.001. The model indicates that patient weight, irradiation time, lab upgrade, and whether PCI was performed are all independent variables influencing patient DAP. Furthermore, time elapsed from 2013 was an independent factor for reduction in patient DAP, suggesting that other factors not included in the model contributed to reduction in patient exposure as time progressed. The complete values are available as supplementary Table 2.

### 3.6. Relationship between Improved Operator Shielding Measures and Operator Dose

A total of 14 operators were active during the analyzed period, including fellows. As data collection ended in June 2019, data for the whole of 2019 were extrapolated.

**Figure 4:**
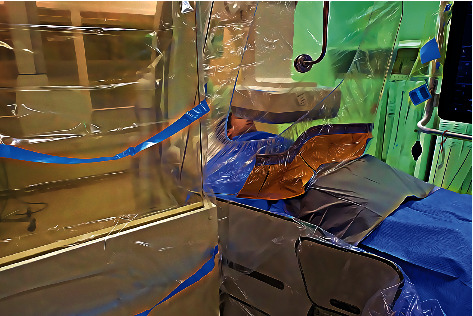
Improved operator shielding setup. Large ceiling-mounted protective shield with panel curtains on the lower end was installed in all cath labs in 2016. Pelvic lead apron was introduced as a standard of care at our center in 2018. Wheel-mounted mobile shield to the left of the operator was used at operator's discretion.

**Figure 5:**
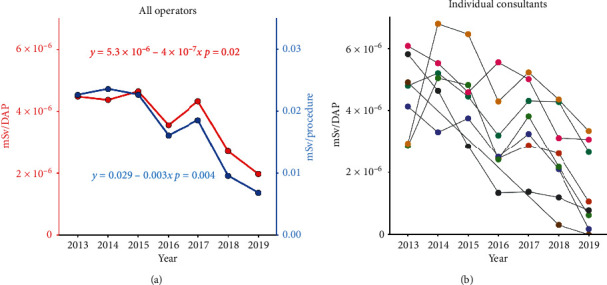
Trends in operator exposure. The ratio between received operator dose and given dose (mSv/DAP) is presented to assess the effects of operator shielding and is not affected by procedure numbers or irradiation time. (a) Pooled trends for mSv/DAP (in red-left, *y*-axis) and mSv/procedure (blue-right, *y*-axis) for all operators and fellows working in the cath lab. Between 2013 and 2019, there was a change in mSv/DAP from 4.48 × 10^−6^ mSv/*μ*Gy·m^2^ to 1.98 × 10^−6^ mSv/*μ*Gy·m^2^, which corresponds to a 56% reduction (*p*=0.02). The reduction in mSv/procedure was larger at 70% (*p*=0.004) as it is influenced by reduced given dose (DAP) per procedure. (b) Interoperator variability between consultants employed throughout the period.

Mean yearly operator dose decreased from 7.5 mSv (range 1.7–20.3) to 2.6 mSv (range 0–5.7) from 2013 to 2019 (supplementary Table 3). To correct for case load and number of operators, the sum of all dosimeter readings of both consultants and fellows was calculated and divided by the total number of procedures performed in the cath lab within each year. The calculated operator dose per procedure showed a 70% reduction from 0.0227 mSv/procedure in 2013 to 0.00685 mSv/procedure in 2019 (*p*=0.004, Table 2). The largest yearly change was observed between 2017 and 2018 (−48%) and coincided with the introduction of improved operator shielding measures (Figure 4). To further investigate the effect of shielding, the ratio between received operator dose (mSv) and patient dose (DAP) was calculated.  Figure 5(a) illustrates the pooled ratio for all operators active in the cath lab including fellows. Between 2013 and 2019, the ratio of operator dose divided by given DAP went from 4.48 × 10^−6^ to 1.98 × 10^−6^ mSv/*μ*Gy·m^2^, which corresponds to a 56% reduction (*p*=0.02). All operators had reduced dosimetry readings per year during the period, but there was a large interoperator variability. In Figure 5(b), the yearly mSv/DAP ratio was calculated separately for the consultants that were active throughout the investigated period in order to illustrate individual changes over time.

## 4. Discussion

Our data show a strong decrease in patient and operator exposure during cardiac catheterization between 2013 and 2019. This finding is likely due to a combination of different factors including new X-ray technology, better operator shielding, and increased awareness.

### 4.1. Patient Characteristics

Our large dataset that covers 21499 procedures performed in our cath lab between 2013 and 2019 shows a trend towards an older, slightly overweight population. The majority of the patients in our data were men that were on average younger than female patients and reflect the known epidemiology of ischemic coronary heart disease [17].

### 4.2. Procedural Characteristics

Between 2013 and 2019, there was a large reduction in patient DAP per procedure, whereas there was only a minor reduction in irradiation time per procedure. Thus, other factors than procedure time such as improved X-ray technologies are likely to explain the observed reduction in DAP per procedure.

### 4.3. Influence of Weight and Age on Irradiation Time and Patient Dose (DAP)

Increased patient weight was correlated to increased patient dose, and doubling of patient weight lead to roughly a three-fold increase in DAP. In the future, the interventional cardiologist is more likely to encounter overweight patients, stressing the importance of better X-ray technology and shielding. Older age was associated with longer irradiation time per procedure, which probably reflects more challenging anatomy and heavily calcified lesions as patient age increases. There was, however, no trend towards increased DAP. The explanation for this is that there is a trend towards lower body weight both in males and females with increasing age. Also, there is a larger proportion of female patients in the older age groups, and females have on average a lower body weight than men.

### 4.4. Impact of the C-Arm Model on Patient Dose (DAP)

X-ray technology is evolving. In the X-ray tube, development of powerful flat emitters that replace conventional helical coils allows for shorter pulse width and better filtration that results in a more efficient photon spectrum. Smaller focal spot size leads to enhanced image sharpness. Detectors with higher dynamic range and bit depth improve image detail accuracy and contrast. Advances in detector technology also include use of thicker scintillators to improve efficiency in conversion of X-rays to image signals. Augmented data processing power allows for software algorithms that enhance image quality and compensates for movement without increased radiation dose. Improved user interface includes default low-dose settings and reduced framerates that can easily be changed by the operator during procedures. All these improvements contribute to more efficient imaging, and our data indicate that newer X-ray systems significantly reduce the radiation required to perform cardiac catheterizations.

### 4.5. Multivariable Analysis of Factors Influencing Patient Dose (DAP)

Reducing patient dose is beneficial for the patient and at the same time reduces operator dose. Multivariable linear regression allows us to evaluate the impact of several factors on patient dose, such as weight, irradiation time, procedure type (PCI or angiography), and lab upgrades. In our analysis, all the aforementioned factors were correlated to patient DAP. Days elapsed since the start of registration was also independently correlated to reduced patient doses, and this suggests that there are other factors not included in the model that have contributed to the reduction in patient dose between 2013 and 2019. Lower frame rates, using more low-dose fluoroscopy instead of high-dose cinefluorangiography, better collimation, and less-angulated projections, are all known to reduce patient dose [18] and are highly operator dependent. The dataset does not contain data about these important parameters, but it is possible that increased awareness and training have led to changes in operator behavior, which have contributed to reduction in patient dose. Our results underscore the importance of an integrated approach that addresses multiple factors influencing patient exposure. Operator training and awareness are crucial to further decrease X-ray doses.

### 4.6. Relationship between Improved Operator Shielding Measures and Operator Dose

Between 2013 and 2019, we observed a marked reduction in mean operator dose per procedure.

The ratio of received operator dose divided by given DAP was calculated as an indicator of operator shielding that is not affected by variations in DAP per procedure. The largest change was between 2017 and 2018 and coincides with the introduction of improved radiation protection. During the introduction of these measures, real-time dosimetry badges were actively used over a period of three months in early 2018. Measurements included dosimetry readings on legs, truncus, and head and helped identify areas for improved radiation shielding. Instant feedback on the effects of radiation reducing behavior likely contributed to increased operator awareness and improved practice.

Although all operators had a trend towards lower radiation exposure, a large interoperator variability was observed. The yearly exposure is of course highly dependent on number of procedures and procedure type. Physicians performing the most complex procedures such as chronic total occlusions are expected to have a higher radiation exposure. However, even when correcting for these factors using the ratio received mSv divided by given DAP, there was still a substantial interoperator variability. This may point to differences in operator behavior and shielding and the possibility for a more focused awareness campaign targeting operators with higher received to given dose ratios.

### 4.7. Implications

Newer X-ray systems with modern detectors significantly reduce the radiation required to produce adequate images, but there is a limit to how much the doses can be lowered without losing vital information in the X-ray images. Thus, simple measures such as reducing fluoroscopic pulse rates, maximal collimation, and optimal position of the patient between the X-ray tube and detector are equally important to reduce patient exposure.

Radio protective garments reduce scatter radiation by >90% in the usual X-ray energy spectra used during coronary angiography [13]. However, they tend to be heavy, uncomfortable, and do not protect operator extremities. Thus, a particularly attractive option is to improve externally mounted shielding that in the future may allow the operators to reduce wearable lead thickness or ideally eliminate its necessity altogether. This may have the added benefit of reducing orthopedic problems and repetitive strain injuries [5, 19]. Our data suggest a significant effect of optimally combining simple available measures as larger ceiling-mounted protective shields with panel curtains, pelvic lead apron, and side shield to eliminate scatter radiating gaps between the patient and operator. Whether such measures may be improved sufficiently to minimize radiation exposure alone or if more sophisticated solutions [20] are required should be further investigated.

### 4.8. Limitations

This is a retrospective, registry-based study, which limits analysis to observations and hypothesis generation. Although mandatory, we do not have the possibility to verify if a personal dosimeter was worn by all operators during all procedures. Radiation protection measures were widely adopted, but we do not have data on the exact percentage, in which pelvic shielding and wheel-mounted side screen was used.

## 5. Conclusion

Our data show a strong trend towards lower patient and operator exposure during percutaneous coronary procedures in the period 2013–2019. Newer X-ray equipment was associated with reduced DAP. The decrease in operator dose was larger than the reduction in DAP, and the largest reduction coincides with the introduction of improved radiation protection measures. This suggests that increased awareness and use of simple external X-ray shielding can have potential to substantially reduce operator radiation exposure.

## Figures and Tables

**Table 1:** Patient characteristics.

	2013	2014	2015	2016	2017	2018	2019	Change 13–19	*P* value
Age in years, mean (median)	66.5 (67)	66.2 (67)	66.7 (68)	66.9 (68)	67.1 (68)	67.4 (69)	67.7 (69)	1.2	0.001
Weight in kg, mean (median)	81.7 (80)	82.3 (81)	81.8 (80)	83 (82)	82.4 (81)	82.6 (82)	83.3 (83)	1.6	<0.001
BMI, mean (median)	27 (26.4)	27.1 (26.6)	27 (26.6)	27.4 (26.7)	27.3 (26.8)	27.3 (26.8)	27.4 (27.1)	0.4	<0.001
Male sex	69.1%	71.3%	69.7%	71.4%	69.8%	70.4%	73.5%	4.4%	NS
Male age in years, mean (median)	65.2 (66)	65 (66)	65.4 (66)	65.7 (67)	65.8 (67)	66.2 (67)	66.7 (68)	1.5	0.002
Female age in year, mean (median)	69.6 (70)	69.3 (70)	69.7 (71)	69.9 (71)	70 (71)	70.5 (72)	70.3 (72)	0.7	0.02
Male weight in kg, mean (median)	86.3 (85)	86.6 (85)	86.6 (85)	87.2 (85)	87 (85)	87.2 (85)	87.7 (86)	1.4	0.003
Female weight in kg, mean (median)	71.2 (70)	71.6 (70)	70.6 (69)	72.5 (71)	71.9 (70)	71.4 (70)	71.4 (70)	0.2	NS
Male height in cm, mean (median)	177.9 (178)	177.8 (178)	177.6 (178)	177.8 (178)	177.7 (178)	177.7 (178)	178 (178)	0.1	NS
Female height in cm, mean (median)	164 (164)	164.2 (164)	164.3 (164)	164.2 (164)	163.6 (164)	163.9 (164)	163.8 (164)	−0.2	NS
Male BMI, mean (median)	27.2 (26.8)	27.4 (26.8)	27.4 (27)	27.5 (26.9)	27.5 (26.9)	27.6 (27.1)	27.7 (27.4)	0.5	<0.001
Female BMI, (median)	26.5 (25.6)	26.5 (26)	26.2 (25.7)	26.9 (26.2)	26.8 (26.3)	26.6 (26.1)	26.6 (25.8)	0.1	NS

*P* values calculated with linear regression for continuous variables and logistic regression for categorical variables with year elapsed as independent variable. BMI = body mass index; kg = kilogram.

**Table 2:** Procedural characteristics.

	2013	2014	2015	2016	2017	2018	2019 (including 30^th^ June)	2019 (projected)	Change 13–19	*P* value
Number of procedures	3318	3268	3210	3275	3372	3348	1708	3444	4%	NS
Number of patients	2915	2922	2851	2935	3057	3015	1564	3154	8%	0.02
Coronary angiography	1934	1837	1661	1664	1807	1777	890	1795	−7%	N. S
PCI	1384	1431	1549	1611	1565	1571	818	1650	19%	0.005
% where PCI was performed	41.7	43.8	48.3	49.2	46.4	46.9	47.9		15%	<0.001
Total operator exposure (mSv)	75.3	77.2	72.9	52.8	62.5	32.1	11.7	23.6	−69%	0.004
Operator exposure per procedure (mSv/procedure)	0.0227	0.0236	0.0227	0.0161	0.0185	0.00959	0.00685	0.00685	−70%	0.004
% valid observations DAP/irradiation time analysis (valid/total)	85.7% (2844/3318)	98.3% (3212/3268)	98.3% (3157/3210)	98.1% (3213/3275)	98.5% (3321/3372)	98.5% (3297/3348)	97.5% (1665/1708)			
Mean irradiation time angiography in seconds (median)	388 (275)	403 (296)	399 (287.5)	377 (277)	343 (240)	344 (253)	353 (246)		−9%	<0.001
Mean irradiation time PCI (median)	1219 (954)	1268 (992)	1200 (957)	1229 (986)	1261 (981)	1135 (890)	1218 (978)		0%	N. S
Mean DAP angio *μ*Gy·m^2^ (median)	2981 (2322)	3091 (2528)	2735 (2203)	2545 (2083)	2431 (1992)	2014 (1592.5)	1891 (1416)		−37%	<0.001
Mean DAP PCI *μ*Gy·m^2^ (median)	8358 (6313.5)	8400 (6856)	7196 (5729)	6583 (5184)	6436 (5157)	5210 (4043)	5055 (4017)		−39%	<0.001
DAP/irradiation time, angio	7.7	7.7	6.9	6.7	7.1	5.9	5.4		−30%	0.003
DAP/irradiation time, PCI	6.9	6.6	6	5.4	5.1	4.6	4.2		−39%	<0.001
DAP/irradiation time, all	7.1	6.9	6.2	5.7	5.6	4.9	4.4		−38%	<0.001

*P* values calculated with linear or logistic regression when appropriate with years elapsed since 2013 as independent variable. DAP = dose area product; mSv = millisievert; PCI = percutaneous coronary intervention; *μ*Gy·*m*2 = microgray squared meters.

## Data Availability

The data used to support the findings of this study are restricted by the Regional Ethics Committee for Medical and Health Research Ethics of Western Norway in order to protect patient privacy. The data are available upon request to the Norwegian Registry for Invasive Cardiology (noric@helse-bergen.no, https://www.kvalitetsregistre.no/registers/norsk-register-invasiv-kardiologi-noric) for researchers who meet the criteria for access to confidential data.

## Supplementary Materials

Supplementary Materials Supplementary table 1: mean and median DAP per procedure according to the C-arm model, installation year, and type of procedure Supplementary table 2: multivariable linear regression evaluating the effects of procedure type (PCI/coronary angiography), days elapsed since the start of 2013, lab upgrades, patient weight, and irradiation time on DAP per procedure Supplementary table 3: dosimetry data of yearly operator exposure in mSv (H10), calculation of mSv per procedure, and mSv per given DAP. Supplementary Figure 1: five plots exploring the relationship between age, weight, sex, irradiation time, and DAP.
