# Supplementary material for: Temporal Trends in X-Ray Exposure during Coronary Angiography and Percutaneous Coronary Intervention
Source: J Interv Cardiol. 2020 Aug 31;2020:9602942. doi: 10.1155/2020/9602942 (PMC7481933; doi:10.1155/2020/9602942)
Supplement: Supplementary Materials — Supplementary table 1: mean and median DAP per procedure according to the C-arm model, installation year, and type of procedure Supplementary table 2: multivariable linear regression evaluating the effects of procedure type (PCI/coronary angiography), days elapsed since the start of 2013, lab upgrades, patient weight, and irradiation time on DAP per procedure Supplementary table 3: dosimetry data of yearly operator exposure in mSv (H10), calculation of mSv per procedure, and mSv per given DAP. Supplementary Figure 1: five plots exploring the relationship between age, weight, sex, irradiation time, and DAP. [file 9602942.f1.docx]

## Supplementary table 1:

## Mean and median DAP per procedure according to C-arm model, installation year and type of procedure

| **Coronary angiography** |  |  |  |  |  |
| --- | --- | --- | --- | --- | --- |
| C-arm | Year installed | mean DAP* | Median DAP* | Lab number | n |
| Philips Azurion7 B12/12 | 2018 | 1650 | 1230 | 1 | 373 |
| Siemens Artis Q | 2016 | 1978 | 1553 | 3 | 2689 |
| Philips Allura Xper FD10C | 2009 | 2742 | 2200 | 2 | 3430 |
| Siemens Axiom Artis dBC | 2006 | 2762 | 2294 | 1 | 3052 |
| Siemens Axiom Artis dFC | 2005 | 3303 | 2630 | 3 | 608 |
| Missing lab number |  | 2920 | 2208 |  | 1024 |
|  |  |  |  |  |  |
| **PCI** |  |  |  |  |  |
| C-arm | Year installed | mean DAP | Median DAP | Lab number | n |
| Philips Azurion7 B12/12 | 2018 | 4798 | 3325 | 1 | 336 |
| Siemens Artis Q | 2016 | 5096 | 4138 | 3 | 2211 |
| Philips Allura Xper FD10C | 2009 | 6924 | 5462 | 2 | 2167 |
| Siemens Axiom Artis dBC | 2006 | 7460 | 5902 | 1 | 3746 |
| Siemens Axiom Artis dFC | 2005 | 8125 | 6832 | 3 | 320 |
| Missing lab number |  | 8410 | 6300 |  | 753 |

*Dose Area Product per procedure expressed in μGy·m2.

## Supplementary table 2:

## Multivariable linear regression evaluating the effects of procedure type (PCI/coronary angiography), days elapsed since start of 2013, lab upgrades, patient weight and irradiation time on DAP per procedure

| Variables | Estimate | 2.5 % | 97.5 % | Pr(>\|t\|) | Std. Error | t value |
| --- | --- | --- | --- | --- | --- | --- |
| Intercept | -4682.5 | -4874.7 | -4490.4 |  | 98 | -47.8 |
| PCI (yes / no) | 794.1 | 710.6 | 877.6 | <0.001 | 42.6 | 18.6 |
| Days elapsed since 01.01.2013 | -0.8 | -0.8 | -0.7 | <0.001 | 0.03 | -24.3 |
| Lab 1 upgrade 2018 | -1044.5 | -1252.8 | -836.2 | <0.001 | 106.3 | -9.8 |
| Lab 3 upgrade 2016 | -776.5 | -870.2 | -682.8 | <0.001 | 47.8 | -16.2 |
| Patient weight in kilograms | 84 | 81.8 | 86.1 | <0.001 | 1.1 | 77.1 |
| Irradiation time in seconds | 4 | 3.9 | 4.1 | <0.001 | 0.03 | 150.3 |

Residual standard error: 2578 on 20480 degrees of freedom. (1012 observations deleted due to missingness)

Multiple R-squared: 0.6856

Adjusted R-squared: 0.6855,

F-statistic: 7442 on 6 and 20480 DF

p-value: < 2.2e-16

## Supplementary table 3:

## Dosimetry data of yearly operator exposure in mSv (H10), calculation of mSv per procedure and mSv per given DAP.

|  | 2013 | 2014 | 2015 | 2016 | 2017 | 2018 | 2019 (30^th^ June) | 2019 * |
| --- | --- | --- | --- | --- | --- | --- | --- | --- |
| Operator 1 | 9.4 | 7.9 | 5.2 | 1.8 | 1.5 | 0.7 | 0.4 | 0.8 |
| Operator 2 | 8.6 |  |  |  |  |  |  |  |
| Operator 3 | 8.3 |  |  |  |  |  |  |  |
| Operator 4 |  | 8.9 | 8.6 | 4.9 | 5.6 | 5.8 | 1.2 | 2.4 |
| Operator 5 | 20.3 | 20.5 | 14.6 | 14.5 | 10.5 | 4.8 | 2.1 | 4.3 |
| Operator 6 | 4.0 | 15.4 | 10.4 | 3.7 | 8.0 | 4.3 | 0.6 | 1.2 |
| Operator 7 |  |  | 0.2 | 8.3 | 14.6 | 1.5 |  |  |
| Operator 8 | 8.0 | 6.9 | 7.0 | 3.5 | 4.6 | 1.8 | 0.1 | 0.2 |
| Operator 9 | 1.7 | 1.6 |  |  |  |  |  |  |
| Operator 10 | 4.6 | 11.6 | 10.5 | 5.0 | 6.5 | 4.2 | 1.6 | 3.3 |
| Operator 11 |  |  | 9.0 | 2.6 |  |  |  |  |
| Operator 12 |  |  |  |  |  |  | 2.8 | 5.7 |
| Operator 13 | 2.0 |  |  |  |  | 0.3 | 0.0 | 0.0 |
| Operator 14 | 8.3 | 4.4 | 7.4 | 8.5 | 11.2 | 8.7 | 2.8 | 5.6 |
|  |  |  |  |  |  |  |  |  |
| **Sum (mSv)** | **75.3** | **77.2** | **72.9** | **52.8** | **62.5** | **32.1** | **11.7** | **23.6** |
|  |  |  |  |  |  |  |  |  |
| **Number of procedures** | 3318 | 3268 | 3210 | 3275 | 3372 | 3348 | 1708 | 3444 |
| **Mean mSv/procedure** | 0.023 | 0.024 | 0.023 | 0.016 | 0.019 | 0.010 | 0.007 | 0.007 |
| **Number of operators** | 10 | 8 | 9 | 9 | 8 | 9 | 9 | 9 |
| **Mean mSv/operator** | 7.5 | 9.7 | 8.1 | 5.9 | 7.8 | 3.6 | 1.3 | 2.6 |
| **Sum DAP**** | 16792342 | 17647703 | 15663593 | 14836041 | 14448598 | 11789061 | 5899311 | 11896401 |
| **mSv per given DAP** | 4.48E-06 | 4.38E-06 | 4.65E-06 | 3.56E-06 | 4.33E-06 | 2.72E-06 | 1.98E-06 | 1.98E-06 |

*Data for whole 2019 are extrapolated from data including 30^th^ June (day 181/365).

** Sum DAP is the sum of all patient DAP for a given year. Missing DAP values were imputed using multiple chained equation imputation

## Supplementary figure 1: Relationship between age, weight, sex, irradiation time and DAP.


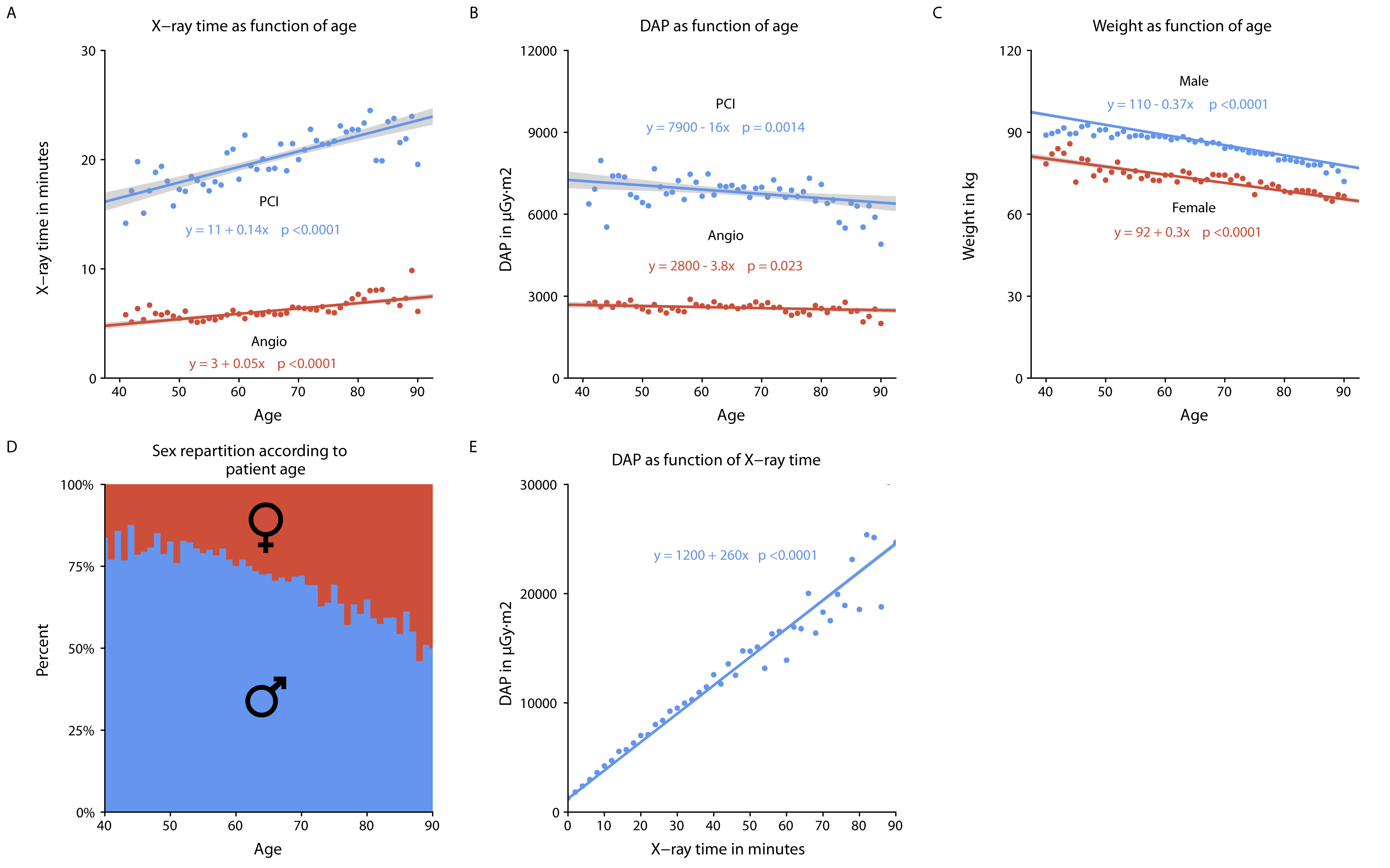


A: Relationship between age and irradiation time. Increase in patient age was associated with longer irradiation time both in coronary angiography and PCI. B: Relationship between age and DAP. Despite a trend towards increased irradiation time in older patients, there was a small trend towards lower DAP with increasing patient age. C: Relationship between age and patient weight. Older patients had lower body weight, both males and females. This contributes to lower DAP in older patients although irradiation times increases. D: Relationship between age and patient sex. Females patients were on average older than men, and thus represent a larger proportion of patients in the older age groups. E: Relationship between DAP and irradiation time. Irradiation time had a strong linear relation to patient
